# Brain volumes and dual-task performance correlates among individuals with cognitive impairment: a retrospective analysis

**DOI:** 10.1007/s00702-020-02199-7

**Published:** 2020-04-29

**Authors:** Jason K. Longhurst, Morgan A. Wise, Daniel J. Krist, Caitlin A. Moreland, Jon A. Basterrechea, Merrill R. Landers

**Affiliations:** 1 Department of Neurorehabilitation, Cleveland Clinic Lou Ruvo Center for Brain Health, 888 West Bonneville, Las Vegas, NV 702-483-6032 USA; 2 Department of Physical Therapy, University of Nevada, Las Vegas, USA; 3 Department of Physical Therapy, University of Nevada, 4505 Maryland Parkway, Box 453029, Las Vegas, NV 702-895-1377 USA

**Keywords:** Dual task interference, Dementia, Cognitive impairment, Brain volume, Cognitive-motor interference

## Abstract

Cognitive impairment (CI) is a prevalent condition characterized by loss of brain volume and changes in cognition, motor function, and dual-tasking ability. To examine associations between brain volumes, dual-task performance, and gait and balance in those with CI to elucidate the mechanisms underlying loss of function. We performed a retrospective analysis of medical records of patients with CI and compared brain volumes, dual-task performance, and measures of gait and balance. Greater cognitive and combined dual-task effects (DTE) are associated with smaller brain volumes. In contrast, motor DTE is not associated with distinct pattern of brain volumes. As brain volumes decrease, dual-task performance becomes more motor prioritized. Cognitive DTE is more strongly associated with decreased performance on measures of gait and balance than motor DTE. Decreased gait and balance performance are also associated with increased motor task prioritization. Cognitive DTE appears to be more strongly associated with decreased automaticity and gait and balance ability than motor DTE and should be utilized as a clinical and research outcome measure in this population. The increased motor task prioritization associated with decreased brain volume and function indicates a potential for accommodative strategies to maximize function in those with CI. Counterintuitive correlations between motor brain volumes and motor DTE in our study suggest a complicated interaction between brain pathology and function.

**Electronic supplementary material:**

The online version of this article (10.1007/s00702-020-02199-7) contains supplementary material, which is available to authorized users.

## Introduction

Dementia is a prevalent problem affecting cognition and function in 46.8 million individuals worldwide, with this number expected to grow to 74.7 million by 2030 and 131.5 million by 2050 (Prince 2015). Dementia care has a tremendous global financial impact, estimated to cost 1 trillion US dollars worldwide and expected to double by 2030 (Prince 2015). Alzheimer’s disease (AD) is the leading cause of dementia and is characterized by cortical atrophy with concomitant memory loss, slowness of performance, and difficulty in performing previously familiar tasks (Jahn 2013; Khan et al. 2014). Other common causes of dementia have changes similar to those found in AD (Jiwa et al. 2010; Vann Jones and O'Brien 2014).

Traditionally, people with dementia are thought to have mainly cognitive deficits; however, these individuals demonstrate motor deficits as well. These include decreased gait speed/quality and balance deficits, which have been associated with fall risk and survival in older adults (Bahureksa et al. 2017; Mirelman et al. 2012; Montero-Odasso et al. 2012; Sheridan and Hausdorff 2007). Additionally, the effects of dementia are seen at the intersection of cognitive and motor function as an impairment of automaticity (Montero-Odasso et al. 2017, 2018; Schwenk et al. 2010). Automaticity is the ability to perform motor and cognitive functions simultaneously without a decline of performance in either task. The automaticity of this dual task (DT) performance has been shown to be significantly decreased in those with AD (Ansai et al. 2017), and slow walking speed during DT gait has been shown to be associated with and predictive of falls, presence of mild cognitive impairment (MCI)—a precursor to AD and other dementias, and the progression of dementia in people MCI (Goncalves et al. 2018; Lowe et al. 2019; MacAulay et al. 2014, 2015,2017; Montero-Odasso et al. 2017).

There is much we do not know about the mechanisms underlying dementia and how they relate to automaticity and other functional deficits. Studies have shown that the cognitive and executive deficits in people with AD are associated with atrophy in specific cognitive-related brain areas such as the frontal lobes and hippocampus (Bruen et al. 2008; Jiji et al. 2013; Laakso et al. 1995). Moreover, the severity and progression of cognitive impairment in other dementias and MCI have also been shown to be related to atrophy and damage in areas such as the hippocampus and striatum (Chen et al. 2015; Jiwa et al. 2010; Mielke et al. 2012; Venkat et al. 2015). However, there is little known about motor relevant brain areas and how they relate to motor and DT function. Likewise, there is little known about the relationship between DT function and cognitive relevant brain areas. Previous studies have identified brain areas uniquely correlated with DT performance, including frontal, temporal, and cingulate regions (Doi et al. 2017; Tripathi et al. 2019). While not the hallmark of cognitive disorders, there is evidence of decreased volume, decrease neuronal integrity, and lower cerebral blood flow in motor-related brain areas such as the basal ganglia and primary motor cortex in those with MCI and dementia, and these findings have been shown to be associated with both single and dual task gait variability (Annweiler et al. 2013; Jiji et al. 2013; Nakamura et al. 1997). Further research is needed to determine if and how the decreased motor and DT function are related to brain volume changes or other factors (Jiji et al. 2013; Tian et al. 2017).

The first aim of this exploratory study was to examine if brain volumes are associated with DT performance in a mixed clinical sample of individuals with cognitive impairment. We hypothesized that motor relevant brain volumes would be more associated with motor dual-task effects (mDTE), indicating a decline in motor performance under DT conditions. Based on the literature, we hypothesized that the superior frontal lobe caudate, putamen, middle parietal and cerebellum would be associated with mDTE (Allali et al. 2019; Poldrack et al. 2005; Tripathi et al. 2019). We also hypothesized that cognitive relevant brain volumes would be more associated with cognitive dual-task effects (cogDTE), indicating a decline in cognitive performance under DT conditions. Based on the literature, we hypothesized that the inferior frontal, hippocampus, and cingulate regions will be associated with cogDTE (Tripathi et al. 2019). The second aim of this study was to determine if task prioritization (i.e., a choice to focus more attention on one of two competing and concurrent tasks, cognitive or motor) related to dual task effect (DTE) can provide information about brain volumes in individuals with cognitive impairment. We hypothesized that individuals who prioritized cognition (i.e., cogDTE decreases less than mDTE) and individuals who prioritized motor function (i.e., mDTE decreases less than cogDTE) would have had different associations with motor and cognitive relevant brain areas. Based on the literature, we hypothesized that level of task prioritization will be associated with volumes in the caudate, anterior cingulate, medial orbital frontal, inferior frontal, superior temporal, and parahippocampal regions (Holtzer et al. 2016; Li et al. 2017; Zheng et al. 2014). The third aim of this study was to determine how general automaticity, as represented by combined dual-task effect (cDTE), was associated with brain regions and gait/balance impairments. We hypothesized that the cDTE would be associated with more brain volumes and gait/balance impairments than either the motor or cognitive DTEs. Based on the literature, with automaticity we hypothesized that superior frontal, inferior frontal, lateral orbitofrontal, caudate, anterior cingulate, entorhinal, and parahippocampal regions would correlate with cDTE (Allali et al. 2019; Li et al. 2017; Poldrack et al. 2005; Sakurai et al. 2019; Tripathi et al. 2019).

## Methods

### Design

A retrospective exploratory analysis of data extracted from medical records for patients diagnosed with memory loss who received physical therapy (PT) treatment at the Cleveland Clinic Lou Ruvo Center for Brain Health (CCLRCBH) from January of 2017 to December of 2018 was conducted. Items that were extracted from the patient medical records included the following: demographic information (ie, sex, age, year of first cognitive symptom, fall history, etc.), diagnoses, cognitive outcome measures, gait and balance outcome measures, and brain volumetric data from MRI. None of the treating physical therapists were involved in the data extraction process. All data were collected under CCLRCBH Institutional Review Board approval.

### Patients

**Fig. 1:**
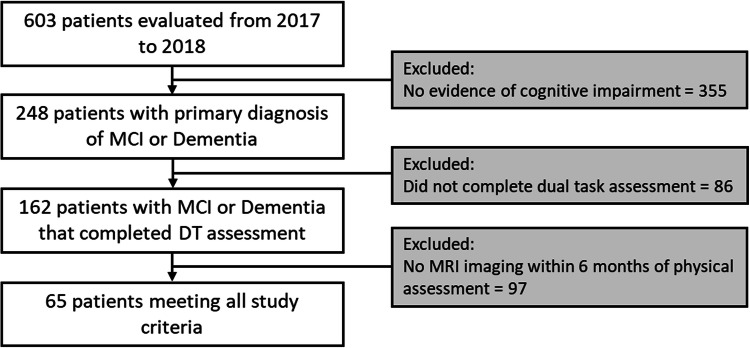
CONSORT flow diagram

All patients with an initial PT evaluation were identified from billing records and screened for inclusion in the study. CCLRCBH considers PT an integral part of memory loss treatment; therefore, patients diagnosed with cognitive impairment or dementia are referred to PT to address motor-related impairments regardless of severity. Clinical diagnosis of disorders of cognition was completed by neurologists using contemporary evidence-based criteria (Albert et al. 2011; McKeith et al. 2017; McKhann et al. 2011; Skrobot et al. 2018, 2017). Patients without data regarding their DT performance or without MRI imaging from within 6 months of DT assessment were excluded. The decision was made to not include individual clinical diagnosis in the analysis primarily due to the uncertain nature of clinical diagnoses, as well as the high prevalence of mixed pathologies. Moreover, mixed diagnoses in analyses like this are not unusual in the dementia literature (Bonner-Jackson et al. 2015; Jones et al. 2019; Lowe et al. 2019; Ritter et al. 2017; Wang et al. 2018; Zink et al. 2018). Therefore, the final data for analysis came from a mixed clinical sample of community dwelling older adults with cognitive impairment (Fig. 1). The general makeup of the patient sample is broadly comparable to known base rates of disease, with clinical diagnoses of AD, dementia with lewy bodies, and cerebrovascular disease being most common, with less common syndromes neurodegenerative disorders (e.g., posterior cortical atrophy, corticobasal syndrome) also seen (Table 1).

**Table 1:** Means, proportions and standard deviations for demographics, dual task effect battery, and measures of cognition, balance, gait, and strength and endurance

	*n*	Mean or proportion
Demographics		
Age (years)	65	76.37 ± 8.83
Sex		
Male	35	53.80%
Female	30	46.20%
Race		
White	51	78.50%
Black	4	6.20%
Asian	4	6.20%
Pacific Islander	1	1.50%
Multiracial	5	7.70%
Ethnicity		
Non-Hispanic	61	93.80%
Hispanic	4	6.20%
Years since first cognitive symptom	61	5.20 ± 3.65
Clinical diagnosis		
Alzheimer’s disease	14	21.50%
Mild cognitive impairment	13	20.00%
Dementia with Lewy bodies	8	12.30%
Vascular cognitive impairment	10	15.40%
Mixed cognitive impairment	6	9.20%
Normal pressure hydrocephalus	2	3.10%
Parkinson’s disease dementia	5	7.70%
Cognitive impairment of unknown etiology	7	10.80%
Falls reported in prior year	58	4.26 ± 12.63
Falls reported in prior 30 days	46	1.15 ± 4.57
Falls injuries reported in prior year	50	0.44 ± 0.91
Dual-task effect battery		
Motor dual task effect	65	44.40 ± 42.52
Cognitive dual task effect	65	114.96 ± 179.79
Attention allocation index	65	− 70.35 ± 175.97
Combined dual task effect	65	226.56 ± 330.44
Cognition		
Montreal cognitive assessment (scale points)	58	19.98 ± 5.81
Balance		
MBT-overall (scale points)	57	18.79 ± 5.97
MBT anticipatory	57	3.98 ± .78
MBT reactive	57	3.76 ± 1.36
MBT sensory organization	57	5.15 ± 1.10
MBT dynamic gait	57	6.07 ± 1.77
Fear of Falling avoidance beliefs questionnaire (scale points)	24	16.17 ± 12.25
Gait		
Ten meter walk test (meters/second)	42	1.00 ± .49
Ten meter walk test – Fast (meters/second)	40	1.47 ± .78
Strength and endurance		
Five times sit to stand (seconds)	59	15.79 ± 7.05
Six minute walk test (meters)	35	325.01 ± 125.37

### Sample size estimation

The sample size was estimated using PASS 15.0.2 (NCSS, LLC. Kaysville, Utah, USA, www.ncss.com/software/pass) and was powered based on aims 1 and 2. For aims 1 and 2, a sample size of 46 would achieve 80% power to detect a Pearson correlation difference of -0.40 between the null hypothesis correlation of 0.00 and the alternative hypothesis correlation of 0.40 using a two-sided hypothesis test with *α* = 0.05.

### Instrumentation

**Brain volumes.** All patients were scanned with the same Siemens Skyra 3 T scanner (Siemens Medical Solutions USA Inc., Malvern, PA, USA). Volumes were measured using NeuroQuant^©^ (CorTechs Labs Inc, La Jolla, California, USA, www.cortechslabs.com/products/neuroquant), an automated program for calculating brain volumes approved by the Food and Drug Administration (Brewer et al. 2009) and good to excellent reliability (Kovacevic et al. 2009; Ochs et al. 2015). The brain areas we considered to be motor relevant were the caudate (Haber 2016; Rosano et al. 2008), putamen (Haber 2016; Rosano et al. 2007, 2008), pallidum (Haber 2016; Rosano et al. 2008), cerebellar white and gray mater (Blumen et al. 2019; Manto et al. 2012; Rosano et al. 2007), brainstem (Blumen et al. 2019; Cedzich et al. 1998), paracentral (Christidi et al. 2018; Rosano et al. 2007), primary motor (Li et al. 2015), primary sensory (Berlucchi and Vallar 2018), medial parietal (Berlucchi and Vallar 2018), superior parietal (Berlucchi and Vallar 2018; Rosano et al. 2007), and inferior parietal (Berlucchi and Vallar 2018; Jubault et al. 2007) regions. The areas we considered to be cognitive relevant were the hippocampus (Duff et al. 2019; Matthews 2015; Opitz 2014; Rosano et al. 2008), amygdala (Sah et al. 2003), transverse and superior temporal (Matthews 2015), posterior superior temporal sulcus (Matthews 2015), middle temporal (Nenciovici et al. 2019), inferior temporal (Matthews 2015; Rosano et al. 2008), fusiform (Matthews 2015; Rosano et al. 2008), parahippocampal (Matthews 2015; Rosano et al. 2008), entorhinal cortex (Matthews 2015; Rosano et al. 2008), temporal pole (Matthews 2015; Rosano et al. 2008), cingulate (Matthews 2015), anterior cingulate (Shenhav et al. 2013), posterior cingulate (Matthews 2015; Rosano et al. 2008), lateral orbitofrontal (Baltz et al. 2018; Rosano et al. 2008), medial orbitofrontal (Baltz et al. 2018), superior frontal (Funahashi and Andreau 2013), inferior frontal (Funahashi and Andreau 2013; Rosano et al. 2008), and nucleus accumbens (Floresco 2015) regions. The data also included whole brain volume, cortical gray matter, and cerebral white matter volumes.

**Dual-task effect-battery (DTE-B).** DT performance was obtained from performance on the Timed Up and Go Test (TUG), and Timed Up and Go—Cognitive (TUGcog), two commonly used clinical tools. For the TUG individuals were instructed to rise from a chair walk 3 m as quickly and safely as possible, across a line marked on the floor, turn around, return to the chair and sit down. The TUGcog was completed in similar manner with the addition of a cognitive task, as described below. Recorded times for the TUG and the TUGcog were used. The TUG exhibits excellent test–retest reliability in individuals with AD (Appendix 1). For community dwelling older adults, the TUGcog has excellent interrater and intrarater reliability (Appendix 1). Prior to performing the TUGcog, individuals performed the cognitive task of serial subtraction by three from a number between 80 and 100 in a seated position to measure their single task (ST) cognitive performance. The number of correct responses in 20 s was recorded and from that was calculated the average number of seconds per correct response, a measure adapted from previous studies that have used correct response rate (Kelly et al. 2010; Yang et al. 2016) Cognitive performance during DT was measured using the same method but beginning at a different number between 80 and 100 to minimize learning effects. Patients were instructed to perform both the motor and cognitive tasks as quickly and accurately as possible. These instructions were intended to encourage equal priority between motor and cognitive tasks. Motor and cognitive DTEs were then calculated using the equation (McIsaac et al. 2015; Yang et al. 2017):

$$\mathrm{D}\mathrm{T}\mathrm{E}\,\left(\mathrm{\%}\right)=\frac{-\mathrm{D}\mathrm{T}-\mathrm{S}\mathrm{T}}{\mathrm{S}\mathrm{T}}\times 100\mathrm{\%}$$

Calculation of cDTEs was designed from the equation for DTE with modifications to include an assessment of the cognitive and motor aspects of the DT interference (Longhurst and Landers 2019), cDTE was calculated using the following equation:

$$\mathrm{c}\mathrm{D}\mathrm{T}\mathrm{E} \,(\%)=\frac{-\left(\mathrm{m}\mathrm{o}\mathrm{t}\mathrm{o}\mathrm{r}\mathrm{D}\mathrm{T} \times \mathrm{c}\mathrm{o}\mathrm{g}\mathrm{n}\mathrm{i}\mathrm{t}\mathrm{i}\mathrm{v}\mathrm{e} \mathrm{D}\mathrm{T}\right)-(\mathrm{m}\mathrm{o}\mathrm{t}\mathrm{o}\mathrm{r}\mathrm{S}\mathrm{T} \times \mathrm{c}\mathrm{o}\mathrm{g}\mathrm{n}\mathrm{i}\mathrm{t}\mathrm{i}\mathrm{v}\mathrm{e}\mathrm{S}\mathrm{T})}{(\mathrm{m}\mathrm{o}\mathrm{t}\mathrm{o}\mathrm{r}\mathrm{S}\mathrm{T} \times \mathrm{c}\mathrm{o}\mathrm{g}\mathrm{n}\mathrm{i}\mathrm{t}\mathrm{i}\mathrm{v}\mathrm{e}\mathrm{S}\mathrm{T})}\times 100\%$$

The cognitive variable is the number of seconds per correct response, which gets larger as performance declines. The motor variable is simply time (in seconds); thus, increased time connotes worse performance. A negative was inserted into the numerator of the equation so that negative DTEs are indicative of a poorer DT performance relative to single task performance (DT cost), while positives DTE are indicative of improved performance under DT conditions relative to single task performance (DT facilitation) (Fritz et al. 2015; Kelly et al. 2010; Plummer and Eskes 2015). Task prioritization, for the second aim, was measured using the modified Attention Allocation Index (mAAI) with a lower value indicating motor prioritization and a higher value indicating cognitive prioritization (Kelly et al. 2010; Siu and Woollacott 2007). mAAI was calculated using the following equation:

$$\mathrm{m}\mathrm{A}\mathrm{A}\mathrm{I}=\mathrm{m}\mathrm{o}\mathrm{t}\mathrm{o}\mathrm{r} \mathrm{D}\mathrm{T}\mathrm{E}-\mathrm{c}\mathrm{o}\mathrm{g}\mathrm{n}\mathrm{i}\mathrm{t}\mathrm{i}\mathrm{v}\mathrm{e} \mathrm{D}\mathrm{T}\mathrm{E}$$

Example data from one participant on these variables (mDTE, cogDTE, mAAI, and cDTE) are included in Appendix 2.

**Cognition.** Scores from the montreal cognitive assessment (MoCA) were used to measure global cognition. The MoCA has excellent test–retest reliability (Appendix 1).

**Balance.** Sensory orientation, anticipatory postural responses, reactive postural control, and dynamic gait were quantified using scores from the Mini-BESTest (MBT), a performance-based balance measure with excellent interrater reliability (Appendix 1). Because patients with a history of falling or imbalance may develop a fear of falling that results in self-imposed activity restrictions, Fear of Falling Avoidance Behavior Questionnaire (FFABQ) scores were used to assess avoidance behavior. The FFABQ has good test–retest reliability and validity in people with neurologic diagnosis (Appendix 1).

**Gait**. Self-selected and fast gait speed from the 10 m Walk Test (10 MWT and 10 MWT-fast) were utilized to measure gait performance. The 10MWT has excellent test–retest reliability (Appendix 1). Similar methods for measuring fast speed have also shown excellent reliability among older adults (Appendix 1).

**Strength and endurance**. Scores from the Six Minute Walk Test (6MWT) and Five Times Sit to Stand Test (5STS) were used to represent walking endurance and lower extremity functional strength, respectively. The 6MWT exhibits excellent test–retest reliability, as well as both interrater and intra-rater reliability for individuals with AD (Appendix 1). The 5STS exhibits excellent test–retest reliability for the community-dwelling elderly (Appendix 1).

### Data analysis

All analyses were conducted using SPSS 24.0 (IBM SPSS Statistics for Windows, Armonk, New York, USA: IBM Corp) with *α* = 0.05 with Benjamini–Hochberg correction for multiple comparisons. For Aim 1 (brain volumes and DTE aim), brain volumetric data were initially compared to mDTE and cogDTE, using Pearson correlation coefficient analyses. To account for potential covariates, hierarchical multiple linear regression analyses were conducted, regressing brain volumes on age, sex, clinical diagnosis, and MoCA in block 1, and then dual take effects (mDTE or cogDTE) in block 2. For aim 2 (brain volumes and task prioritization aim), mAAI was used to determine associations with brain volumes using Pearson correlation coefficients. To account for potential covariates hierarchical multiple linear regression analyses were conducted, regressing brain volumes on age, sex, clinical diagnosis, and MoCA in block 1, and then mAAI in block 2. For Aim 3 (DTE, brain volumes, and physical performance aim), brain volumetric data were compared to cDTE, using Pearson correlation coefficient analyses. To account for potential covariates heirarchal multiple linear regression analyses were conducted, regressing brain volumes on age, sex, clinical diagnosis, and MoCA in block 1, and dual take effects (mDTE, cogDTE, or cDTE) in block 2. The strength of relationships between brain volumes and mDTE, cogDTE, and cDTE were compared using Pearson correlation coefficients. The strength of relationships between measures of gait (preferred gait speed, fast gait speed, timed up and go test, and TUGcog), balance (MBT, BBS, and FFABQ), and strength and endurance (6MWT and 5STS) and mDTE, cogDTE, and cDTE were also compared using Pearson correlation coefficients.

## Results

**Fig. 2:**
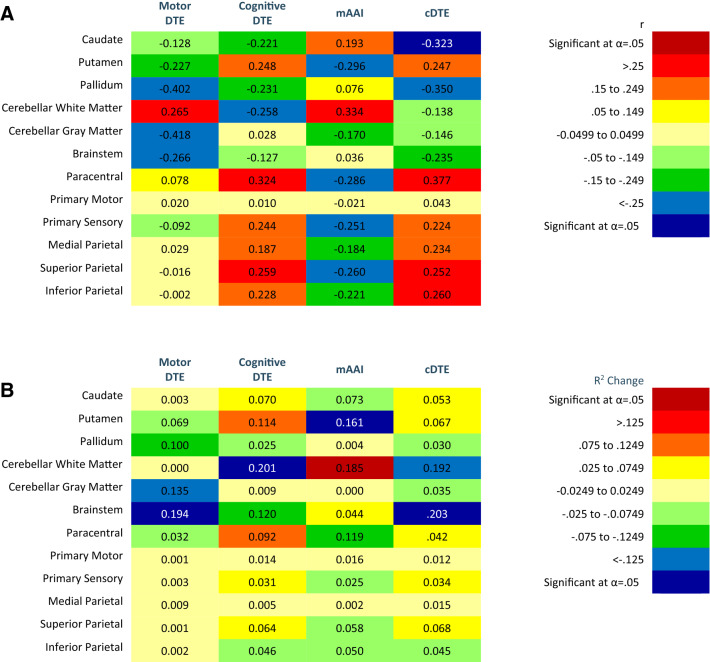
Heat maps of motor relevant brain areas on motor DTE, cognitive DTE, modified attention allocation index, and cDTE. **a** Person’s correlations. **b***R*^2^ Change of hierarchal regression, regressing motor relevant brain volumes on dual task performance controlling for age, sex, diagnosis, and Montreal Cognitive assessment. Color (cool or warm) determined by positive or negative regression coefficient value. Brain volumes analyzed as percent of inter-cranial volume. Significant findings as indicated after Benjamini–Hochberg procedure

**Fig. 3:**
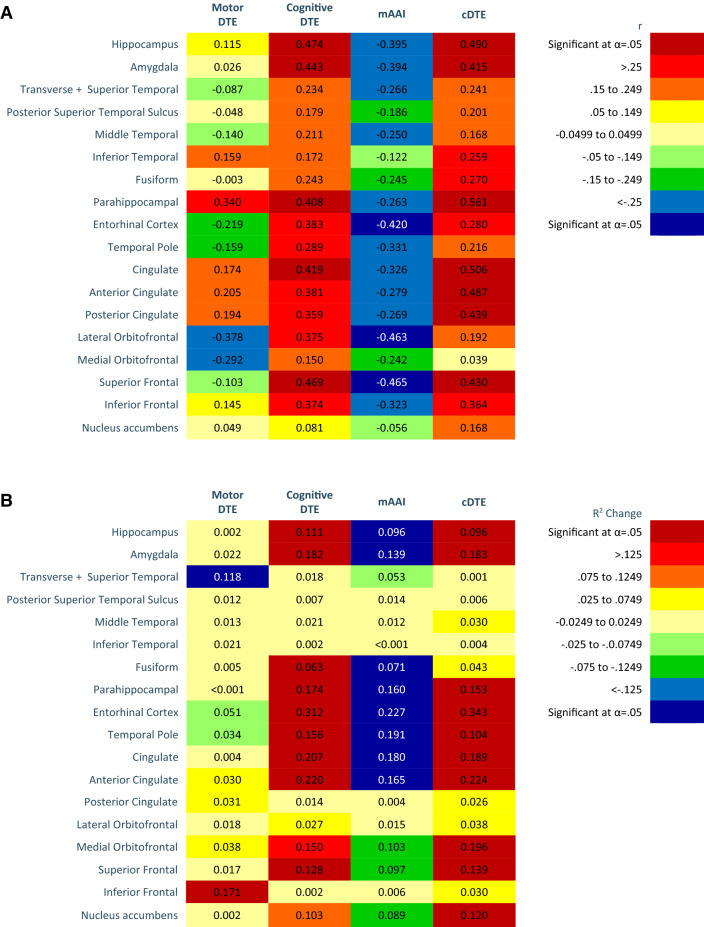
Heat maps of cognitive relevant brain areas on motor DTE, cognitive DTE, modified attention allocation index, and cDTE. **a** Person’s correlations (*r*). **b***R*^2^ Change of hierarchal regression, regressing cognitive relevant brain volumes on dual task performance controlling for age, sex, diagnosis, and Montreal Cognitive assessment. Color (cool or warm) determined by positive or negative regression coefficient value. Brain volumes analyzed as percent of inter-cranial volume. Significant findings as indicated after Benjamini–Hochberg procedure

**Fig. 4:**
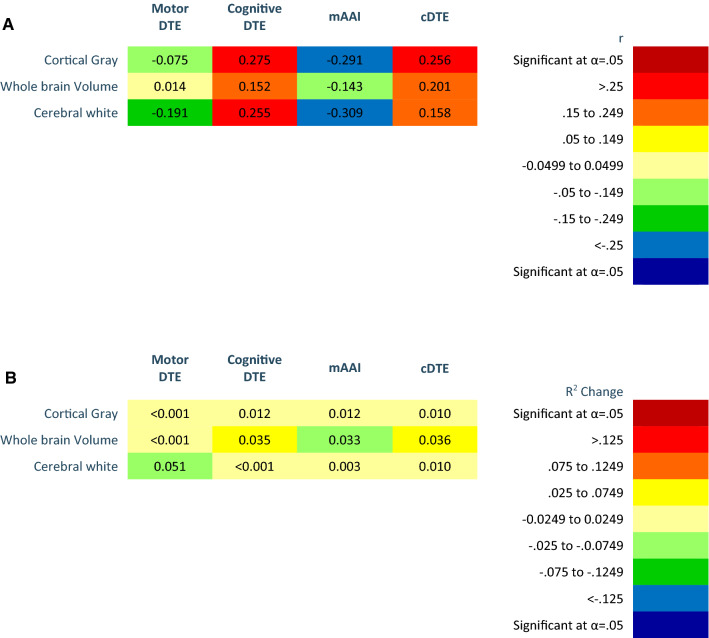
Heat maps of whole brain relevant brain areas on motor DTE, cognitive DTE, modified attention allocation index, and cDTE. **a** Person’s correlations. **b***R*^2^ Change of hierarchal regression, regressing whole brain volumes on dual task performance controlling for age, sex, diagnosis, and Montreal Cognitive assessment. Color (cool or warm) determined by positive or negative regression coefficient value. Brain volumes analyzed as percent of inter-cranial volume. Significant findings as indicated after Benjamini–Hochberg procedure

**Aim 1 (brain volumes and DTE aim).** Correlations, *R*^2^ changes, probability values, and unstandardized (*b*) and standardized (*β*) regression of coefficients of motor relevant brain volumes and DTE are found in Fig. 2 and Appendix 3. For the heat maps (Figs. 2, 3, 4), warm colors are associated with a positive correlation between motor brain volume and DTE. Cool colors are associated with an inverse relationship between motor brain volume and DTE. Heat maps of the correlations and *R*^2^ change for cognitive relevant brain volumes and DTE are found in Fig. 3, and for whole brain volumes and DTE in Fig. 4. Probability values and unstandardized (*b*) and standardized (*β*) regression of coefficients are reported in Appendix 4 and 5, respectively. More negative cogDTE, or a greater decrement in performance during cognitive performance during DT, was associated with decreased volume in both motor and cognitive relevant brain regions. While mDTE was not consistently associated with cognitive relevant brain volumes, more negative mDTE was associated with slightly larger motor brain areas.

**Aim 2 (brain volumes and task prioritization aim).** Correlations and *R*^2^ changes of task prioritization and brain volumes are coded in Figs. 2, 3, 4 using heat maps. Probability values and unstandardized (*b*) and standardized (*β*) regression of coefficients are reported in Appendices 3–5. A higher value mAAI represents motor prioritization; therefore, an inverse correlation indicates that smaller brain volumes are associated with motor prioritization. Our results indicate that as brain volumes decreased DT performance became more motor prioritized.

**Fig. 5:**
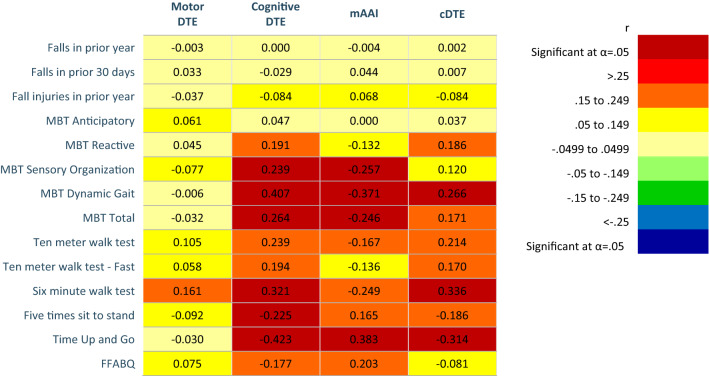
Heat map of measures of gait and balance on motor DTE, cognitive DTE, attention allocation index, and cDTE. Since a higher value on some gait and balance measures means better performance and a lower value on others means better performance, we have coded the proper relationship with the color rather than the positive or negative values in the table. Abbreviation key: MBT (Mini-BESTest), FFABQ (Fear of Falling Avoidance Behavior Questionnaire). Significant findings as indicated after Benjamini–Hochberg procedure

**Fig. 6:**
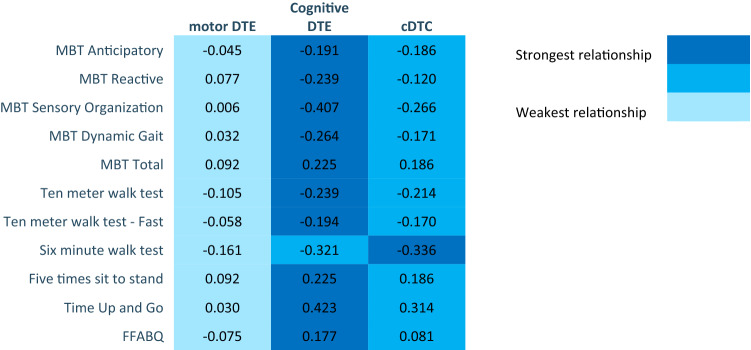
Heat map comparing strength of relationship on measures of gait and balance measures among motor DTE, cognitive DTE, and cDTE. Abbreviation key: MBT (Mini-BESTest), FFABQ (Fear of Falling Avoidance Behavior Questionnaire)

**Aim 3 (DTE, brain volumes, and physical performance aim).** Correlations and *R*^2^ changes of cDTE and brain volumes are coded in Figs. 2, 3, 4 using heat maps. Probability values and unstandardized (*b*) and standardized (*β*) regression of coefficients are reported in Appendices 3–5. Correlations of DTE and task prioritization on gait and balance measures are contained in heat maps in Fig. 5, with probability values reported in Appendix 6. To determine which of the DTEs (cognitive, motor, and combined) are most associated with measures of gait and balance, a heat map was created to show the strongest and weakest associations (Fig. 6). The pattern of association of the cDTE and brain volumes was similar to cogDTE alone. Decreased gait and balance performance were associated with greater motor task prioritization. CogDTE is more strongly associated with performance on measures of gait and balance compared to mDTE.

## Discussion

**Motor relevant brain volumes and DTE.** More negative mDTE (representing greater loss of motor performance during DT) was associated with larger brain volumes in motor relevant areas (Fig. 2a). These associations persisted after statistically controlling for covariates (age, sex, clinical diagnosis, and MoCA), but were limited primarily to cerebellar gray mater, and the brain stem (Fig. 2b). This is counterintuitive as one would expect decreased motor performance to be associated with atrophy of motor areas. Fischer et al. found a greater mDTE to be associated with decreased caudate volume in those with AD and MCI, but did not consider other motor areas or investigate other components of the DTE-B (Fischer et al. 2017). They also found an association between higher cerebellar gray matter volume and worse performance on the TUG. This is consistent with the association we observed between cerebellar gray matter and mDTE and reinforces the counterintuitive conclusion that larger brain volumes may be associated with decreased motor performance during DT in this population. In contrast to our findings, Annweiler et al. found decreased motor cortex volume to be associated with slower gait speeds during both single and DT gait in those with MCI, and found no association with other regions (Annweiler et al. 2013). It is unclear why larger motor brain volumes would be correlated with worse mDTE, but relative preservation of motor structures may affect resource allocation when there are competing motor and cognitive demands during DT performance.

More negative cogDTE, representing a decline in cognitive performance during DT, was associated with a decrease in motor-related brain volumes (Fig. 2a). After controlling for covariates, the pattern of associations became less clear with the strongest associations being with cerebellar white mater and the brainstem. It could be that if these regions are relatively spared (Kunst et al. 2019), they are called upon to compensate for loss of function in other areas and thus contributes less to their original function. Rosano et al. found no association between gait parameters and the cerebellum (Rosano et al. 2008). This may suggest a complicated role of the cerebellum, especially when applied to DT processing. There were also notable positive correlations, including the putamen and paracentral lobules. The paracentral lobule is interesting as it has been implicated in integrating multisensory information, and has been shown to be involved in body perception and voluntary attention (Daprati et al. 2010). We would anticipate DT paradigms to probe this region and, with its role in integrating sensory information with body awareness and driving attention, it is logical that it would be associated with cogDTE. Further research in this area is warranted.

Motor-relevant brain volumes were inversely associated with mAAI, which persisted after controlling for covariates; however, the strength of the relationships were lower (Fig. 2a, b). This indicates that atrophy in motor brain areas was associated with increasing motor prioritization. The lone exception was cerebellar white matter. Motor prioritization during DT means the individual either mostly maintains their gait speed, has significantly poorer cognitive performance, or both. This could be the result of an intentional focus on gait for safety despite the neutral instructions, or an unconscious default strategy to prioritize gait. Other researchers have suggested that individuals with limited attentional resources may adopt a “posture first” strategy of prioritizing gait during DT to avoid falling (Plummer and Eskes 2015; Yogev-Seligmann et al. 2012). Individuals with AD have even been found to increasingly prioritize postural control during a DT as the difficulty of the postural control component increases, which suggests that DT training may be utilized to reduce fall risk by manipulating the parameters of the component tasks (Rapp et al. 2006).

When we look at cDTE, representing the combined cognitive and motor DTE, we see a similar pattern with that of cogDTE for motor-relevant brain areas (Fig. 2a, b). This indicates that the cognitive loss during DT may be the primary driver of loss of automaticity and DT performance among individuals with cognitive impairment. Notably, the associations with cDTE and most relevant regions are diminished after controlling for covariates, particularly MoCA scores. It may be that regions included in our analysis of motor regions have overlapping and significant cognitive functions. This may particularly be the case for the parietal lobes which consistently signaled moderate relationships with both cogDTE and cDTE; however, these findings diminished upon controlling for cognition (with the exception of the paracentral lobule). The parietal lobe has significant visuospatial function which has been shown to be a hallmark deficit in AD and other dementias (Mandal et al. 2012). It can be detected early in the disease process and is associated with disease progression and global cognitive function (Lam et al. 2013; Mandal et al. 2012). Considering our sample was a mixed clinical sample, our findings suggest that the parietal lobe findings are mostly a function of cognitive ability. Overall, our results suggest that that poorer DT performance may be associated with larger volumes in the brain stem and cerebellar white mater, and may be linked to smaller volumes in the putamen and paracentral lobules. However, overall brain volumes in motor regions did not demonstrate a consistent pattern of association with DT performance.

**Cognitive-relevant brain volumes and DTE.** There does not appear to be any consistent pattern of association between mDTE and cognitive brain volumes (Fig. 3a, b). We did observe a greater decrement in DT cognitive performance as cognitive-brain area volume decreased. This is logical and consistent with research that has demonstrated that cognitive deficits in this population are associated with atrophy of cognitive brain areas and with the notion that cognitive regions play a key role in performance of motor-cognitive DTs (Bruen et al. 2008; Laakso et al. 1995).

We found that decreased brain volume in cognitive areas was associated with motor task prioritization (Fig. 3a, b). Since dementia is characterized by atrophy of cognitive-related brain areas, this indicates that the more advanced a person’s cognitive impairment, the more they prioritize motor function during DT. Motor prioritization may be a useful adaptation in those with cognitive impairment, since it could minimize fall risk during activities requiring DT. Notably, even healthy older adults tend to prioritize walking when a demanding secondary task is added (Li et al. 2001; Maclean et al. 2017). In contrast to our findings, Theill et al. (2011) showed that healthy older adults tend to motor prioritize while those with cognitive impairment tend to prioritize cognition. However, in contrast, that study excluded individuals with severe cognitive impairment and utilized an easier cognitive task. Research on this topic has been inconsistent, with some studies showing healthy older adults tend to prioritize cognition during a DT (Corp et al. 2018; Schaefer et al. 2015). It is generally acknowledged that response to DT conditions is context dependent and can be altered by the characteristics of the individual, the task, and the environment (Shumway-Cook et al. 1997).

We also observed that cDTE was associated with decreased volume in cognitive brain areas in a similar pattern to cogDTE. This indicates that most of the overall loss of automaticity is driven by a loss of cognitive function. Notably the regions that showed the strongest association with cogDTE, mAAI and cDTE were the hippocampus, amygdala, fusiform, parahippocampal region, temporal poles, anterior cingulate, medial orbital frontal regions, and superior frontal regions. As a group these regions fairly closely match the stated hypothesis drawn from literature. Further functional imaging studies are warranted to investigate the specific roles of these regions in dual task performance and automaticity. In general, our findings on cognitive-relevant brain volumes are consistent with other research showing that decreased volume is associated with a worse performance during DT in those with cognitive impairment (Doi et al. 2017). They also highlight the utility of DT performance metrics beyond mDTE as has been suggested in other populations (Longhurst and Landers 2019; Plummer and Eskes 2015; Plummer et al. 2013; Yang et al. 2017).

**Whole brain volumes and DTE.** After controlling for covariates, we found no pattern of association in whole brain volumes with DT performance (Fig. 4b). Extensive patterns of gray matter involvement have been associated with DT performance in those with cognitive impairment and would be expected to drive whole brain patterns (Doi et al. 2017); however, our findings do not support this notion. Our results support the notion that the majority of the variation seen in whole brain volumes is a function of global cognitive function.

**Physical performance and DTE**. We found little to no association between mDTE and gait and balance in those with cognitive impairment (Fig. 5a, b). This is notable because mDTE is a common clinical outcome measure and the most commonly used measure in research on the effect of DT gait in those with AD (Fritz et al. 2015). It is known that decline in motor function can be one the earliest and best predictors of progression to dementia (Montero-Odasso et al. 2018). Our study did find cogDTE to be associated with decreased performance in almost all measures of gait and balance, which indicates that cogDTE may be a better representation of global function than mDTE in those with cognitive impairment.

Motor task prioritization appears to be associated with decreased gait and balance. In light of our brain volume observations, this suggests that those with worse cognitive impairment have worse gait and balance and more motor prioritization during DT. As noted previously, a “posture first” framework suggest that individuals prioritize gait over a cognitive task when fall avoidance has a more immediate perceived value (Shumway-Cook et al. 1997). Other researchers have suggested that task prioritization depends on factors such as the individual’s perception of postural hazards and capacity to respond to postural destabilization (Yogev-Seligmann et al. 2012). Adults with decreased perceptual/motor capabilities, such as those seen in cognitive impairment, have less capacity to respond to postural threats and may utilize more cognitive resources to compensate, thereby incurring a greater cogDTE (Yogev-Seligmann et al. 2012). However, individuals can demonstrate a maladaptive “posture second” strategy if they are unable to accurately perceive postural hazards (Bloem et al. 2006).

cDTE was also correlated with decreased gait and balance performance, and shows a similar pattern to cogDTE. Combined with the lack of correlations for mDTE, this reinforces the notion that cogDTE may be the driving factor in the loss of DT performance in this population. It is worth noting that different causes of cognitive impairment have different underlying brain changes and different patterns of brain activation during DT (Maclean et al. 2017). It may be challenging to generalize about DT performance and the implications in our population, as the trends that we found may not hold true for all types and causes of cognitive impairment.

Interestingly, fall history was not associated with loss of DT ability in our sample, indicating that DT performance may not be a good identifier of fall risk among individuals with cognitive impairment. Other studies have found conflicting results; finding DT performance effective for detecting fall risk (Muir et al. 2012), while others have found DT performance to be no more effective than single task performance (Taylor et al. 2013); and still others have found no association between falls and DT performance (McCulloch et al. 2010). DT impairments have been shown to be associated with fall risk in community dwelling older adults in general (Muir-Hunter and Wittwer 2016), but it may differ for those with cognitive impairment specifically. It could be that those with cognitive impairment decrease their activity level and exposure to situations in which falls may occur, either secondary to cognitive deficits or intentionally due to fear of falling, and balance and functional deficits are therefore not reflected in fall history.

CogDTE was most strongly associated with measures of gait and balance in our sample (Fig. 6), while mDTE was least associated with measures of gait and balance. The exception was the 6MWT, which was more strongly associated with cDTE. This makes sense, because cDTE is a representation of automaticity, and a six-minute ambulation task is more automatic in character than the other measures (Huxhold et al. 2006). It could be that cogDTE is more relevant than cDTE for most clinical outcome measures where tasks are discrete, short in duration, and unfamiliar to the patient.

**Limitations.** Our study utilized a mixed clinical sample of cognitive impairment. Different causes of CI can have different underlying disease processes and may have showed different correlations individually. This warrants further investigation. Additionally, we analyzed the data categorizing each brain region by its primary function, being either cognitive or motor, and there is evidence that many brain regions have both motor and cognitive functions. While we did select brain regions based on the literature, the analyses were exploratory and our results should be interpreted with caution. More hypothesis driven studies are warranted to confirm these findings including looking a specific cognitive impairment diagnoses.

## Conclusion

Overall, our results suggest several interesting patterns and directions for future research. First, the cognitive cost of DT appears to be more prominent and the primary driver for lost automaticity when compared to the motor cost in those with cognitive impairment. This is notable because clinicians and researchers have often used change in gait speed during DT as the primary, if not the only, outcome measure for DT ability in this population. While mDTE may be seen as more relevant due to potential associated fall risk and functional limitation, cogDTE may have a stronger association with gait and balance ability in this population and should not be overlooked when assessing function and response to treatment. Second, with decreased volume of cognitive related brain areas, those with dementia appear to increasingly prioritize the motor component of a DT. In other words, as dementia progresses, individuals tend to sacrifice cognition while concentrating on their gait. This indicates a potential for accommodative strategies in this population that could be exploited through neuro-rehabilitation interventions to maximize function. Finally, while most of the brain volume and DTE correlations in our results are intuitive and expected, some are not. The inverse correlation between the increased volume of motor-relevant brain areas and decline in DT performance suggests a more complicated interaction between specific brain regions, disease processes, and function in those with cognitive impairment and warrants further research.

## Electronic supplementary material

Below is the link to the electronic supplementary material.

Supplementary file1 (DOCX 22 kb)

Supplementary file2 (DOCX 12 kb)

Supplementary file3 (DOCX 47 kb)

## Abbreviations

10MWT: Ten meter walk test
10MWT-fast: Ten meter walk test–fast
5STS: Five times sit to stand test
6MWT: Six minute walk test
AD: Alzheimer’s disease
CCLRCBH: Cleveland Clinic Lou Ruvo Center for Brian Health
cDTE: Combined dual task effect
CI: Cognitive impairment
cogDTE: Cognitive dual task effect
DT: Dual task
DTE: Dual task effect
FFABQ: Fear of falling avoidance behavior questionaire
mAAI: Modified attention allocation index
MBT: MiniBESTest
MCI: Mild cognitive impairment
mDTE: Motor dual task effect
MoCA: Montreal cognitive assessment
PT: Physical therapy
ST: Single task
TUG: Timed up and go
TUGcog: Timed up and go–cognitive
