# Supplementary material for: Brain volumes and dual-task performance correlates among individuals with cognitive impairment: a retrospective analysis
Source: J Neural Transm (Vienna). 2020 Apr 29;127(7):1057–71. doi: 10.1007/s00702-020-02199-7 (PMC7293667; doi:10.1007/s00702-020-02199-7)
Supplement: Supplementary file 1 — Supplementary file1 (DOCX 22 kb) [file 702_2020_2199_MOESM1_ESM.docx]

**Appendix 1.** Table of reliability statistics of outcome measures utilized.

|  | | **Reliability Statistic** |
| --- | --- | --- |
| **DUAL TASK EFFECT BATTERY** | |  |
|  | Timed up and Go | Test-retest: ICC=0.99^1^ |
|  | Timed Up and Go - Cognitive | Intra-rater: ICC=0.94^2^, Inter-rater: 0.99^3^ |
| **COGNITION** | |  |
|  | Montreal Cognitive Assessment | Test-retest: correlation coefficient=.92^4^ |
| **BALANCE** | |  |
|  | Berg Balance Scale | Test-retest: Cronbach’s α = 0.77^5^, Inter-rater: ICC=0.87^5^ |
|  | MiniBESTest | Inter-rater: ICC=0.87^6^ |
|  | Fear of Falling Avoidance Beliefs Questionnaire | Test-retest: ICC=0.81^7,8^ |
| **GAIT** | |  |
|  | Ten meter walk test | Test-retest: ICC=0.98^9^ |
|  | Ten meter walk test - Fast | Test-retest: ICC=0.96^10^ |
| **STRENGTH AND ENDURANCE** | |  |
|  | Five times sit to stand | Test-retest: ICC=0.96^11^ |
|  | Six minute walk test | Test-retest: ICC=0.98^1^, Intra-rater: ICC=0.83^12^, Inter-rater: ICC=0.98^12^ |

References:

1. Ries JD, Echternach JL, Nof L, Gagnon Blodgett M. Test-retest reliability and minimal detectable change scores for the timed "up & go" test, the six-minute walk test, and gait speed in people with Alzheimer disease. *Phys Ther.* 2009;89(6):569-579.

2. Hofheinz M, Schusterschitz C. Dual task interference in estimating the risk of falls and measuring change: a comparative, psychometric study of four measurements. *Clin Rehabil.* 2010;24(9):831-842.

3. Shumway-Cook A, Brauer S, Woollacott M. Predicting the probability for falls in community-dwelling older adults using the Timed Up & Go Test. *Phys Ther.* 2000;80(9):896-903.

4. Nasreddine ZS, Phillips NA, Bedirian V, et al. The Montreal Cognitive Assessment, MoCA: a brief screening tool for mild cognitive impairment. *J Am Geriatr Soc.* 2005;53(4):695-699.

5. Wang CY, Hsieh CL, Olson SL, Wang CH, Sheu CF, Liang CC. Psychometric properties of the Berg Balance Scale in a community-dwelling elderly resident population in Taiwan. *J Formos Med Assoc.* 2006;105(12):992-1000.

6. Godi M, Franchignoni F, Caligari M, Giordano A, Turcato AM, Nardone A. Comparison of reliability, validity, and responsiveness of the mini-BESTest and Berg Balance Scale in patients with balance disorders. *Phys Ther.* 2013;93(2):158-167.

7. Landers MR, Durand C, Powell DS, Dibble LE, Young DL. Development of a scale to assess avoidance behavior due to a fear of falling: the Fear of Falling Avoidance Behavior Questionnaire. *Phys Ther.* 2011;91(8):1253-1265.

8. Landers MR LM, Newman M. Reliability and validity of the modified Fear of Falling Avoidance Behavior Questionnaire in Parkinson’s disease. . *Movement Disorders.* 2014;29:177-178.

9. Peters DM, Fritz SL, Krotish DE. Assessing the reliability and validity of a shorter walk test compared with the 10-Meter Walk Test for measurements of gait speed in healthy, older adults. *J Geriatr Phys Ther.* 2013;36(1):24-30.

10. Marchetti G, Hodges M, Brown R, Krohn K. TEST-RETEST RELIABILITY, EXTERNAL STRUCTURE VALIDITY AND RESPONSIVENESS OF GAIT PARAMETERS FOR OLDER ADULT FEMALES WALKING AT PREFERRED AND MAXIMUM VELOCITY. *Journal of Geriatric Physical Therapy.* 2005;28(3):114.

11. Bohannon RWS, Megan E. | Barreca, Susan R. | Masters, Lisa M. | Sigouin, Christopher S. Five-repetition sit-to-stand test performance by community-dwelling adults: A preliminary investigation of times, determinants, and relationship with self-reported physical performance. *Isokinetics and Exercise Science.* 2007;15(2):77-81.

12. Tappen RM, Roach KE, Buchner D, Barry C, Edelstein J. Reliability of physical performance measures in nursing home residents with Alzheimer's disease. *J Gerontol A Biol Sci Med Sci.* 1997;52(1):M52-55.
