# Supplementary material for: Brain volumes and dual-task performance correlates among individuals with cognitive impairment: a retrospective analysis
Source: J Neural Transm (Vienna). 2020 Apr 29;127(7):1057–71. doi: 10.1007/s00702-020-02199-7 (PMC7293667; doi:10.1007/s00702-020-02199-7)
Supplement: Supplementary file 2 — Supplementary file2 (DOCX 12 kb) [file 702_2020_2199_MOESM2_ESM.docx]

**Appendix 2.** Example participant data for dual task effect battery.

|  | **Abbreviation** | **Example data** |
| --- | --- | --- |
| *Timed Up and Go* | TUG or STmotor | 10.00 seconds |
| *Seated correct response rate* | STcog | 2 seconds/ correct response |
| *Timed Up and Go – Cognitive* | TUG-cog |  |
| *Dual task motor performance* | DTmotor | 13.50 seconds |
| *Dual task cognitive performance* | DTcog | 3 seconds/correct response |
| *Motor dual task effect* | mDTE | -35.00% |
| *Cognitive dual task effect* | cogDTE | -50.00% |
| *Modified attention allocation index* | mAAI | 15.00% |
| *Combined dual task effect* | cDTE | -102.5% |
