# Supplementary material for: Brain volumes and dual-task performance correlates among individuals with cognitive impairment: a retrospective analysis
Source: J Neural Transm (Vienna). 2020 Apr 29;127(7):1057–71. doi: 10.1007/s00702-020-02199-7 (PMC7293667; doi:10.1007/s00702-020-02199-7)
Supplement: Supplementary file 3 — Supplementary file3 (DOCX 47 kb) [file 702_2020_2199_MOESM3_ESM.docx]

**Appendix 3. A**. Pearson correlation and probability values for motor relevant brain areas on motor DTE, cognitive DTE, modified attention allocation index, and cDTE. **B.** Unstandardized (b) coefficients, (standardized (β) coefficients), and probability values of R2 change of hierarchal regression of motor relevant brain volumes on DT performance controlling for age, sex, diagnosis, and Montreal cognitive assessment. Values marked in red indicate significant after Benjamini-Hochberg procedure. Brain Volumes analyzed as percents of inter-cranial volume.

| **A** | **Motor**  **DTE** | **Cognitive**  **DTE** | **mAAI** | **cDTE** |
| --- | --- | --- | --- | --- |
| Caudate | 0.128  p=0.308 | 0.221  p=0.081 | -0.193  p=0.130 | 0.323  p=0.001 |
| Putamen | 0.227  p=0.171 | -0.248  p=0.123 | 0.296  p=0.064 | -0.247  p=0.124 |
| Pallidum | 0.402  p=0.012 | 0.231  p=0.152 | -0.076  p=0.642 | 0.350  p=0.027 |
| Cerebellar White Matter | -0.265  p=0.107 | 0.258  p=0.109 | -0.334  p=0.035 | 0.138  p=0.396 |
| Cerebellar Gray Matter | 0.418  p=0.009 | -0.028  p=0.862 | 0.170  p=0.294 | 0.146  p=0.367 |
| Brainstem | 0.266  p=0.107 | 0.127  p=0.434 | -0.036  p=0.826 | 0.235  p=0.145 |
| Paracentral | -0.078  p=0.644 | -0.324  p=0.042 | 0.286  p=0.074 | -0.377  p=0.016 |
| Primary Motor | -0.020  p=0.906 | -0.010  p=0.950 | 0.021  p=0.896 | -0.043  p=0.793 |
| Primary Sensory | 0.092  p=0.584 | -0.244  p=0.129 | 0.251  p=0.119 | -0.224  p=0.165 |
| Medial Parietal | -0.029  p=0.861 | -0.187  p=0.247 | 0.184  p=0.254 | -0.234  p=0.146 |
| Superior Parietal | 0.016  p=0.922 | -0.259  p=0.107 | 0.260  p=0.106 | -0.252  p=0.117 |
| Inferior Parietal | 0.002  p=0.990 | -0.228  p=0.158 | 0.221  p=0.171 | -0.260  p=0.105 |

| **B** | **Motor**  **DTE** | **Cognitive**  **DTE** | **mAAI** | **cDTE** |
| --- | --- | --- | --- | --- |
| Caudate | .036 (.069)  p=0.731 | -.058 (-.358)  p=0.054 | .058 (.373)  p=0.048 | -.032 (-.296)  p=0.099 |
| Putamen | .169 (0.350)  p=0.061 | -.065 (-.455)  p=0.016 | .076 (.552)  p=0.003 | -.032 (-.335)  p=0.073 |
| Pallidum | .172 (.422)  p=0.013 | .028 (.212)  p=0.323 | -.010 (-.083)  p=0.705 | .019 (.255)  p=0.270 |
| Cerebellar White Matter | .017 (.029)  p=0.877 | .101 (.606)  p<.001 | -.095 (-.592)  p=0.001 | .062 (.567)  p=0.396 |
| Cerebellar Gray Matter | .290 (.488)  p=0.023 | .023 (.127)  p=0.555 | .002 (.014)  p=0.948 | .029 (.243)  p=0.233 |
| Brainstem | .367 (.586)  p=0.004 | .086 (.469)  p=0.025 | -.051 (-.289)  p=0.190 | .070 (.582)  p=0.002 |
| Paracentral | .149 (.237)  p=0.282 | -.076 (-.409)  p=0.063 | .084 (.475)  p=0.033 | -.032 (-.266)  p=0.217 |
| Primary Motor | .015 (.030)  p=0.882 | -.024 (-.161)  p=0.444 | .025 (.177)  p=0.410 | -.014 (-.144)  p=0.475 |
| Primary Sensory | -.048 (.073)  p=0.648 | -.047 (-.239)  p=0.117 | .041 (.219)  p=0.161 | -.031 (-.240)  p=0.100 |
| Medial Parietal | -.089 (-.124)  p=0.548 | -.021 (-.097)  p=0.621 | .012 (.060)  p=0.766 | -.022 (-.156)  p=0.405 |
| Superior Parietal | -.024 (-.036)  p=0.865 | -.067 (-.342)  p=0.086 | .063 (.331)  p=0.103 | -.044 (-.336)  p=0.077 |
| Inferior Parietal | .032 (.056)  p=0.990 | -.049 (-.291)  p=0.152 | .050 (.307)  p=0.138 | -.030 (-.267)  p=0.157 |

**Appendix 4.** Pearson correlation and probability values for cognitive relevant brain areas on motor DTE, cognitive DTE, modified attention allocation index, and cDTE. **B.** Unstandardized (b) coefficients, (standardized (β) coefficients), and probability values of R2 change of hierarchal regression of cognitive relevant brain volumes on DT performance controlling for age, sex, diagnosis, and Montreal cognitive assessment. Values marked in red indicate significant after Benjamini-Hochberg procedure. Brain Volumes analyzed as percents of inter-cranial volume.

| **A** | **Motor**  **DTE** | **Cognitive DTE** | **mAAI** | **cDTE** |
| --- | --- | --- | --- | --- |
| Hippocampus | -0.115  p=0.493 | -0.474  p=0.002 | 0.395  p=0.012 | -0.490  p<.001 |
| Amygdala | -0.026  p=0.879 | -0.443  p=0.004 | 0.394  p=0.012 | -0.415  p=0.008 |
| Transverse + Superior Temporal | 0.087  p=0.605 | -0.234  p=0.146 | 0.266  p=0.097 | -0.241  p=0.134 |
| Posterior Superior Temporal Sulcus | 0.048  p=0.774 | -0.179  p=0.269 | 0.186  p=0.252 | -0.201  p=0.214 |
| Middle Temporal | 0.140  p=0.403 | -0.211  p=0.191 | 0.250  p=0.121 | -0.168  p=0.300 |
| Inferior Temporal | -0.159  p=0.341 | -0.172  p=0.288 | 0.122  p=0.451 | -0.259  p=0.107 |
| Fusiform | 0.003  p=0.983 | -0.243  p=0.131 | 0.245  p=0.128 | -0.270  p=0.092 |
| Parahippocampal | -0.340  p=0.037 | -0.408  p=0.009 | 0.263  p=0.101 | -0.561  p<.001 |
| Entorhinal Cortex | 0.219  p=0.186 | -0.383  p=0.015 | 0.420  p=0.007 | -0.280  p=0.080 |
| Temporal Pole | 0.159  p=0.341 | -0.289  p=0.071 | 0.331  p=0.037 | -0.216  p=0.180 |
| Cingulate | -0.174  p=0.295 | -0.419  p=0.007 | 0.326  p=0.040 | -0.506  p<0.001 |
| Anterior Cingulate | -0.205  p=0.217 | -0.381  p=0.015 | 0.279  p=0.081 | -0.487  p<0.001 |
| Posterior Cingulate | -0.194  p=0.244 | -0.359  p=0.023 | 0.269  p=0.093 | -0.439  p=0.005 |
| Lateral Orbitofrontal | 0.378  p=0.019 | -0.375  p=0.017 | 0.463  p=0.003 | -0.192  p=0.235 |
| Medial Orbitofrontal | 0.292  p=0.076 | -0.150  p=0.355 | 0.242  p=0.133 | -0.039  p=0.812 |
| Superior Frontal | 0.103  p=0.503 | -0.469  p=0.002 | 0.465  p=0.003 | -0.430  p=0.006 |
| Inferior Frontal | -0.145  p=0.385 | -0.374  p=0.018 | 0.323  p=0.042 | -0.364  p=0.021 |
| Nucleus accumbens | -0.049  p=0.771 | -0.081  p=0.618 | 0.056  p=0.730 | -0.168  p=0.300 |

| **B** | **Motor**  **DTE** | **Cognitive DTE** | **mAAI** | **cDTE** |
| --- | --- | --- | --- | --- |
| Hippocampus | -.041 (-.057)  p=0.567 | -.099 (-.450)  p<0.001 | .091 (.428)  p<0.001 | -.058 (-.400)  p<.0.001 |
| Amygdala | -.124 (-.195)  p=0.109 | -.115 (-.576)  p<0.001 | .098 (.513)  p<0.001 | -.072 (-.553)  p<0.001 |
| Transverse + Superior Temporal | .287 (.457)  p=0.011 | -.034 (-.180)  p=0.338 | .058 (.318)  p=0.091 | -.006 (-.048)  p=0.790 |
| Posterior Superior Temporal Sulcus | .092 (.145)  p=0.458 | -.021 (-.115)  p=0.551 | .029 (.161)  p=0.412 | -.013 (-.104)  p=0.574 |
| Middle Temporal | -.085 (-.151)  p=0.163 | -.033 (-.198)  p=0.058 | .024 (.151)  p=0.164 | -.024 (-.223)  p=0.023 |
| Inferior Temporal | -.099 (-.192)  p=0.255 | -.009 (-.059)  p=0.722 | <.001 (.001)  p=0.994 | -.009 (-.086)  p=0.584 |
| Fusiform | .064 (.096)  p=0.494 | -.068 (-.339)  p=0.006 | .071 (.367)  p=0.003 | -.035 (-.270)  p=0.027 |
| Parahippocampal | -.012 (.018)  p=0.895 | -.117 (-.563)  p<0.001 | .110 (.550)  p<0.001 | -.069 (-.506)  p<.001 |
| Entorhinal Cortex | -.188 (-.300)  p=0.049 | -.143 (-.755)  p<0.001 | .119 (.657)  p<0.001 | -.094 (-.757)  p<0.001 |
| Temporal Pole | .118 (.247)  p=0.077 | -.075 (-.533)  p<0.001 | .081 (.602)  p<0.001 | -.038 (-.417)  p=0.002 |
| Cingulate | -.059 (-.083)  p=0.698 | -.138 (-.614)  p=0.001 | .126 (.584)  p=0.002 | -.083 (-.562)  p=0.001 |
| Anterior Cingulate | -.155 (-.231)  p=0.227 | -.132 (-.634)  p<0.001 | .112 (.560)  p=0.003 | -.083 (-.611)  p<0.001 |
| Posterior Cingulate | -.156 (-.234)  p=0.032 | -.032 (-.160)  p=0.134 | .017 (.091)  p=0.413 | -.027 (-.209)  p=0.037 |
| Lateral Orbitofrontal | -.095 (-.180)  p=0.208 | -.036 (-.224)  p=0.109 | .026 (.167)  p=0.247 | -.026 (-.252)  p=0.057 |
| Medial Orbitofrontal | -.140 (-.258)  p=0.306 | -.084 (-.524)  p=0.029 | .068 (.441)  p=0.076 | -.060 (-.573)  p=0.011 |
| Superior Frontal | -.074 (-.172)  p=0.418 | -.062 (-.483)  p=0.013 | .053 (.428)  p=0.034 | -.041 (-.483)  p=0.009 |
| Inferior Frontal | -.246 (-.550)  p<0.001 | -.007 (-.054)  p=0.745 | -.014 (-.108)  p=0.552 | -.019 (-.224)  p=0.149 |
| Nucleus accumbens | -.041 (-.064)  p=0.765 | -.085 (-.433)  p=0.025 | .077 (.411)  p=0.039 | -.058 (-.449)  p=0.015 |

**Appendix 5.** **A.** Pearson correlation and probability values for whole brain areas on motor DTE, cognitive DTE, modified attention allocation index, and cDTE. **B.** Unstandardized (b) coefficients, (standardized (β) coefficients), and probability values of R2 change of hierarchal regression of whole brain volumes on DT performance controlling for age, sex, diagnosis, and Montreal cognitive assessment. Values marked in red indicate significant after Benjamini-Hochberg procedure. Brain Volumes analyzed as percents of inter-cranial volume.

| **A** | **Motor**  **DTE** | **Cognitive DTE** | **mAAI** | **cDTE** |
| --- | --- | --- | --- | --- |
| Cortical gray mater | 0.075  p=0.653 | -0.275  p=0.086 | 0.291  p=0.068 | -0.256  p=0.111 |
| Whole brain | -0.014  p=0.918 | -0.152  p=0.278 | 0.143  p=0.308 | -0.201  p=0.150 |
| Cerebral white mater | 0.191  p=0.250 | -0.255  p=0.112 | 0.309  p=0.052 | -0.158  p=0.329 |

| **B** | **Motor**  **DTE** | **Cognitive DTE** | **mAAI** | **cDTE** |
| --- | --- | --- | --- | --- |
| Cortical gray mater | .002 (.003)  p=0.985 | -.029 (-.150)  p=0.392 | .028 (.152)  p=0.394 | -.017 (-.132)  p=0.430 |
| Whole brain | <.001 (.001)  p=0.994 | -.008 (-.259)  p=0.140 | .007 (.244)  p=0.156 | -.005 (-.240)  p=0.135 |
| Cerebral white mater | .132 (.300)  p=0.112 | .002 (.018)  p=0.922 | .009 (.072)  p=0.707 | .011 (.131)  p=0.461 |

**Appendix 6.** Pearson correlation and probability values for measures of gait and balance on motor DTE, cognitive DTE, modified attention allocation index (motor – cognitive), and cDTE. Significant correlations after Benjamini-Hochberg procedure marked in red. Abbreviation key: MBT (Mini-BESTest), FFABQ (Fear of Falling Avoidance Behavior Questionnaire).

|  | **Motor**  **DTE** | **Cognitive DTE** | **mAAI** | **cDTE** |
| --- | --- | --- | --- | --- |
| Falls in prior year | 0.003  p=0.975 | 0.000  p=0.955 | 0.004  p=0.964 | -0.002  p=0.980 |
| Falls in prior 30 days | -0.033  p=0.731 | 0.029  p=0.773 | -0.044  p=0.658 | -0.007  p=0.943 |
| Fall injuries in prior year | 0.037  p=0.674 | 0.084  p=0.352 | -0.068  p=0.453 | 0.084  p=0.353 |
| MBT Anticipatory | -0.061  p=0.498 | -0.047  p=0.617 | 0.000  p=0.999 | -0.037  p=0.151 |
| MBT Reactive | -0.045  p=0.617 | -0.191  p=0.041 | 0.132  p=0.160 | -0.186  p=0.046 |
| MBT Sensory Organization | 0.077  p=0.395 | -0.239  p=0.010 | 0.257  p=0.006 | -0.120  p=0.202 |
| MBT Dynamic Gait | 0.006  p=0.951 | -0.407  p<0.001 | 0.371  p<0.001 | -0.266  p=0.004 |
| MBT Total | 0.032  p=0.721 | -0.264  p=0.004 | 0.246  p=0.008 | -0.171  p=0.066 |
| Ten meter walk test | -0.105  p=0.261 | -0.239  p=0.014 | 0.167  p=0.088 | -0.214  p=0.029 |
| Ten meter walk test - Fast | -0.058  p=0.545 | -0.194  p=0.054 | 0.136 p=0.180 | -0.170  p=0.093 |
| Six minute walk test | -0.161  p=0.140 | -0.321  p=0.004 | 0.249  p=0.026 | -0.336  p=0.002 |
| Five times sit to stand | 0.092  p=0.266 | 0.225  p=0.008 | -0.165  p=0.053 | 0.186  p=0.029 |
| Time Up and Go | 0.030  p=0.699 | 0.423  p<0.001 | -0.383  p<0.001 | 0.314  p<0.001 |
| FFABQ | -0.075  p=0.534 | 0.177  p=0.154 | -0.203  p=0.102 | 0.081  p=0.520 |
